# Hydrological features and the ecological niches of mammalian hosts delineate elevated risk for Ross River virus epidemics in anthropogenic landscapes in Australia

**DOI:** 10.1186/s13071-018-2776-x

**Published:** 2018-03-20

**Authors:** Michael G. Walsh, Cameron Webb

**Affiliations:** 1 Marie Bashir Institute for Infectious Diseases and Biosecurity, Westmead Institute for Medical Research, University of Sydney, Westmead, New South Wales Australia; 2 Department of Medical Entomology, NSW Health Pathology, Westmead Hospital, Westmead, New South Wales Australia

**Keywords:** Ross River virus, Reservoir host, Mosquito-borne, Hydrology, Australia

## Abstract

**Background:**

The current understanding of the landscape epidemiology of Ross River virus (RRV), Australia’s most common arthropod-borne pathogen, is fragmented due to gaps in surveillance programs and the relatively narrow focus of the research conducted to date. This leaves public health agencies with an incomplete understanding of the spectrum of infection risk across the diverse geography of the Australian continent. The current investigation sought to assess the risk of RRV epidemics based on abiotic and biotic landscape features in anthropogenic landscapes, with a particular focus on the influence of water and wildlife hosts.

**Methods:**

Abiotic features, including hydrology, land cover and altitude, and biotic features, including the distribution of wild mammalian hosts, were interrogated using a Maxent model to discern the landscape suitability to RRV epidemics in anthropogenically impacted environments across Australia.

**Results:**

Water-soil balance, proximity to controlled water reservoirs, and the ecological niches of four species (*Perameles nasuta*, *Wallabia bicolor*, *Pseudomys novaehollandiae* and *Trichosurus vulpecula*) were important features identifying high risk landscapes suitable for the occurrence of RRV epidemics.

**Conclusions:**

These results help to delineate human infection risk and thus provide an important perspective for geographically targeted vector, wildlife, and syndromic surveillance within and across the boundaries of local health authorities. Importantly, our analysis highlights the importance of the hydrology, and the potential role of mammalian host species in shaping RRV epidemic risk in peri-urban space. This study offers novel insight into wildlife hosts and RRV infection ecology and identifies those species that may be beneficial to future targeted field surveillance particularly in ecosystems undergoing rapid change.

**Electronic supplementary material:**

The online version of this article (10.1186/s13071-018-2776-x) contains supplementary material, which is available to authorized users.

## Background

Ross River virus (RRV) causes greater human morbidity than any other vector-borne pathogen in Australia. This alphavirus accounts for approximately 5100 reported cases per year nationwide [1], approximately ten times as many human infections as all other zoonoses combined [2]. The epidemiology of RRV transmission is complex and involves multiple reservoir hosts and a diverse range of mosquito vectors [3]. Moreover, these hosts and vectors exploit varied ecological niches across Australia and thus have the potential to modulate significant heterogeneity in enzootic, epizootic and zoonotic transmission of RRV. As such, the landscape epidemiology and infection ecology of RRV, particularly with respect to human spillover and the emergence of subsequent epidemics, remains incomplete despite several decades of state-centralized surveillance across Australia [1].

There are over 40 mosquito species that have been implicated in the transmission of RRV through field detection of isolates or laboratory vector competence experiments [4, 5]. While relatively few of these mosquitoes may actually play a significant role in driving epidemics, substantial variation in the biology and ecology of distinct species can influence their habitat associations, host-feeding preferences, population dynamics and propensity to bite humans [6]. Moreover, wildlife hosts, acting as either viral reservoirs or amplifiers, are a cornerstone of RRV infection ecology. In particular, the distribution of important macropod species such as the eastern grey kangaroo (*Macropus giganteus*) and the western grey kangaroo (*Macropus fuliginosus*), have been identified as important reservoir hosts [7, 8], while in peri-urban spaces the common brushtail possum (*Trichosurus vulpecula*) has been implicated [9]. Nevertheless, the extent to which individual species influence transmission dynamics across broad geography and heterogeneous landscapes is unknown, as is their interaction with those abiotic features of the landscape known to influence vector ecology such as climate, land cover and surface water.

Previously, small localized studies have investigated RRV risk associated with some of these abiotic factors, but these are typically in response to individual epidemics, and often evaluate such factors, especially weather events, in isolation and without consideration of wildlife hosts. For example, precipitation and temperature have frequently been identified as important mediators of human epidemics, but are generally considered outside the broader milieu of abiotic and biotic influence [10–17]. Moreover, non-climatic abiotic mediators have been apportioned relatively little consideration in the study of epizootic and epidemic RRV. Nevertheless, landscape features such as surface water [18–20] and littoral dynamics [21] may exert strong influences on RRV occurrence since these play important roles in shaping the population dynamics of both vector mosquitoes and reservoir hosts. The geometry of abiotic features coincident with human populations and anthropogenic environmental change may be critical in delineating the shape of risk in the landscape [22], particularly in urban environments where specific land use may influence the local distribution of reservoir hosts and mosquito populations [23]. The current investigation applied a machine learning approach to the analysis of ProMED electronic surveillance system data to assess the influence of hydrology and the ecological niches of wild mammalian hosts in delineating RRV landscape suitability, while also accounting for climate, altitude and land cover.

## Methods

The International Society of Infectious Diseases’ ProMED-mail electronic surveillance system was used to identify 51 reports of epidemic RRV in humans across Australia from 1 January, 1996 to 1 July, 2016 (http://www.promedmail.org/). All ProMED-mail reports are vetted by registered health professionals with areas of expertise relevant to the event being reported and located geographically as close as possible to the event [24]. The system comprises 59 appointed moderators, correspondents, and editors who are available to review the reports as they are generated. In addition, there are currently more than 70,000 subscribers in at least 185 countries who are available to comment on epidemic reports for a further level of vetting within the broader infectious diseases community. Notwithstanding a near real-time process of evaluation by a committed body of professionals, the system relies on signals generated not by formal standardized methods of active data collection, but rather by the social propagation of information, which is highly influenced by human population distribution and infrastructure. As such, it is important to note that any surveillance data generated from such a system necessarily represent a particular cross-section of disease occurrence rather than a representative sample of all experience. Nevertheless, the coverage of zoonotic epidemics by the ProMED-mail system has been shown to be good in Australia [25]. For the current study, this means that RRV epidemics captured by ProMED are a sample of large events occurring in landscapes of significant human influence. Sporadic cases will be missed entirely, and even epidemics that represent a large increase relative to the baseline occurrence may also be missed if they occur in small populations. As such, while we must emphasize that the scope of this study does not apply to the full spectrum of human RRV experience, we do correct for potential reporting bias (see statistical methods below), which allows for an unbiased assessment of RRV landscape suitability in anthropogenic environments. The geographical coordinates for the location of each RRV epidemic were referenced in Google Maps and cross-referenced in Open Street Map. Five of the 51 documented epidemics were reported with a spatial resolution greater than five kilometers or without geographical information and, therefore, were excluded from the analysis. The final analytic sample comprised 46 epidemics across the twenty-year study period.

The geographical distributions of RRV host species were based on observed specimens obtained from the Global Biodiversity Information Facility (GBIF) (http://www.gbif.org/). Species distribution models were constructed for *Macropus giganteu*s (*n* = 31,293), *M. fuliginosis* (*n* = 8844), *M. rufus* (*n* = 15,940), *M. robustus* (*n* = 17,998), *M. agilis* (*n* = 1840), *M. rufogriseus* (*n* = 27,568), *M. parryi* (*n* = 264), *Wallabia bicolor* (*n* = 347,082), *Trichosurus vulpecula* (*n* = 1754), *Isoodon obesulus* (*n* = 4563), *Perameles nasuta* (*n* = 82), *Pteropus poliocephalus* (*n* = 2421), *Pteropus alecto* (*n* = 1144), *Hydromys chrysogaster* (*n* = 840), *Rattus sordidus* (*n* = 75) and *Pseudomys novaehollandiae* (*n* = 993), all of which have previously been identified by serology or viral isolation as RRV hosts [5, 7–9, 26–30]. Only observations recorded over the same period as the occurrence of RRV epidemics (1996–2016) were included to maintain the temporal continuity between species distribution models and the landscape suitability model of RRV epidemics. All mammal specimens included here were restricted to the spatial extent of latitude 54.74973–10.05167°S, and longitude 159.1019–112.9511°E. With these field samples, Maxent models (see the description of the statistical methods in the following section) were used to model their ecological niches.

The human footprint (HFP) was quantified using data obtained from Socioeconomic Data and Applications Center (SEDAC) [31]. The HFP was calculated in two stages. First, the human influence index (HII) was constructed. The HII measures the impact of human presence on the landscape as a function of eight domains: (i) population density; (ii) proximity to railroads; (iii) proximity to roads; (iv) proximity to navigable rivers; (v) proximity to coastlines; (vi) intensity of nighttime artificial light; (vii) location in or outside delineated urban space; and (viii) land cover. The domains are scored according to the level of human impact per geographical unit, whereby higher scores signify greater human influence. A composite index is then created by combining the eight individual domains. This composite ranges from 0, indicating an absence of human influence (i.e. a parcel of land unaltered by human activity), to 64, indicating maximal human influence in the landscape. The HII composite is subsequently normalized according to the 15 terrestrial biomes defined by the World Wildlife Fund to obtain the HFP. The normalization is represented as a ratio of the range of minimum and maximum HII in each biome to the range of minimum and maximum HII across all biomes and is expressed as a percentage with a spatial resolution of approximately 1 km^2^ [32]. A measure of human migration from 1990 to 2000 was also obtained from SEDAC and was derived from the Global Rural-Urban Mapping Project estimates for the year 2000 [33]. Net human migration over this period was represented as the net population flow into and out of each 1 km^2^ area, where positive and negative values indicate net gains and losses, respectively [34, 35].

Climate data aggregated over the period 1950 to 2000 were obtained from the WorldClim Global Climate database [36] with the following data products used in this investigation: mean temperatures for the hottest and coldest quarters, and mean precipitation for the wettest and driest quarters. In addition to the climate data, a raster for altitude was also obtained from this database. Each of these five data products was extracted as a 1 km^2^ resolution raster [37].

Vegetation cover was assessed using the MODIS-based Maximum Green Vegetation Fraction (MGVF), which is a data product from the United States Geologic Survey's Land Cover Institute [38]. The MGVF records the percentage of green vegetation cover per pixel as a function of the normalized difference vegetation index at a resolution of 1 km^2^ [39]. The MGVF raster represented the average vegetation cover of each year between 2001 and 2012.

Raster data for the distributions of surface water were obtained from the Global Lakes and Wetlands Database (http://www.worldwildlife.org/pages/global-lakes-and-wetlands-database) at a resolution of 1 km^2^ each. This raster was derived from three distinct data levels, two of which were vector format and one raster. Level 1 consisted of the polygons of all lakes with area ≥ 50 km^2^ and reservoirs with volume ≥ 0.5 km^3^. Level 2 comprised the polygons of all surface water with area ≥ 0.1 km^2^. Level 3 combined the first two levels and rasterized the vector data while also adding more information on wetlands to the final data product [40]. Each surface water type was extracted and a new raster isolating each feature created. Subsequently, the distance between each pixel and the nearest pixel of each unique surface water feature was calculated to create a discrete distance raster for each land cover and water type. All distances were calculated in the QGIS geographical information system using the proximity procedure. This allowed for a more nuanced approach to modeling proximity to landscape features rather than crudely assigning presence *versus* absence of features to each 1 km^2^ area (see modeling description below). Distance rasters for the following surface water types were represented in the analysis: lake, reservoir, river, freshwater wetland, coastal wetland and intermittent wetland.

To interrogate the movement of water through the landscape and its relationship to RRV epidemics, two hydrological data products were acquired from the Hydrological Data and Maps based on SHuttle Elevation Derivatives at multiple Scales (HydroSHEDS) information system (https://hydrosheds.cr.usgs.gov/), which is derived from elevation data of the Shuttle Radar Topography Mission [41]. First, hydrological flow accumulation was obtained as a 15 arc-second raster and measures the quantity of upstream area draining into each 500 × 500 m area, and second, a 15 arc-second raster of all stream networks. We also obtained the position of all controlled water reservoirs and dams in Australia from the Global Reservoir and Dam Database [42, 43], which is maintained by SEDAC. As a final assessment of hydrological risk, we used the Priestley-Taylor alpha coefficient (P-Tα) as a robust indicator of water-soil balance in the landscape [44, 45]. This coefficient is the ratio of actual evapotranspiration to potential evapotranspiration and represents water stress in each 1 km^2^ by capturing both water availability in the soil and water requirements of the local vegetation. Thus, the measure is a robust estimate of environmental water stress through soil-water balance. A global raster for P-Tα was retrieved from the Consultative Group for International Agricultural Research (CGIAR) Consortium for Spatial Information. The ratio is dimensionless and ranges from 0 to 1 but was scaled to 0 to 100 for easier interpretation [46].

### Statistical analysis

This investigation applied the machine learning algorithm, Maxent, to modeling and mapping the suitability of heterogeneous Australian landscapes to RRV epidemics. This algorithm estimates the density of predictor variables conditional on observed occurrences to model the landscape suitability of those occurrences [47, 48]. Moreover, Maxent has wide global application to modeling the ecological niches of many zoonotic infectious diseases [49–51]. Maxent has also been shown to be one of the most effective approaches to modeling landscape suitability, performing better than both other machine learning algorithms and more conventional statistical models including generalized linear models [51, 52].

Maxent models were employed in this study to (i) classify and map separately the ecological niche of RRV host species, and (ii) subsequently describe the landscape suitability for RRV epidemics. Presence points are represented by the documented species occurrences, or epidemic RRV events, respectively, while background points were selected within the geographical extent of latitude 54.74973–10.05167°S, and longitude 159.1019–112.9511°E. Reporting bias is an important consideration in ecological niche modeling as occurrences of species (or cases of disease) are more likely to be observed or recorded in areas or contexts that are more accessible. To account for such bias in our target species observations, and reporting bias of RRV epidemics in humans, background points were sampled proportional to the HFP (using a unique sampling for each species niche), which incorporates population density as well as other developed infrastructure [53]. Ten thousand background points were sampled to model epidemic RRV landscape suitability.

The modeling proceeded in two steps. First, output raster maps of the RRV wildlife host niches were created from the ecological niche models and then subsequently used to model RRV epidemics. The Maxent models used for the host niches included mean temperature during the warmest and coolest quarters, mean precipitation during the wettest and driest quarters, MGVF, and human migration as predictors of reservoir habitat suitability at a 1 km^2^ resolution.

Secondly, these species distribution models were used in the subsequent model predicting RRV epidemic suitability. The Maxent model for RRV epidemics included the ecological niches of the wildlife hosts, proximity to each surface water type, proximity to controlled water reservoirs, water-soil balance (P-Tα), hydrological flow accumulation, altitude, and MGVF. Correlation among the landscape factors was generally low (Pearson's correlation coefficients < 0.6) with the exception of precipitation and *M. giganteus*, which were highly correlated with many of the factors in the RRV landscape suitability model and therefore not included in the model. Nevertheless, we are confident minimal information derived from precipitation would have been lost since the P-Tα coefficient was included, which accounts for water availability due to precipitation in its calculation. Model predictions were presented as landscape suitability, expressed as a percentage. All Maxent models (for reservoirs and RRV epidemics) were cross-validated using 5-fold cross-validation with the cross-validation mean area under the curve (AUC) reported as a percentage.

Landscape features used in the Maxent models were ranked according to their permutation importance, which randomly permutes the values of the landscape factors between background and presence points and is more robust to any residual correlation among the features and therefore preferred over the potentially more naïve percent contribution to the loss function [47, 54].

All analyses were conducted in the R environment [55], with the exception of the computation of the distance rasters, which, as described above, was conducted using QGIS. The maxent function in the *dismo* package (v. 0.9–3) was used to fit the models [54, 56].

## Results

**Fig. 1:**
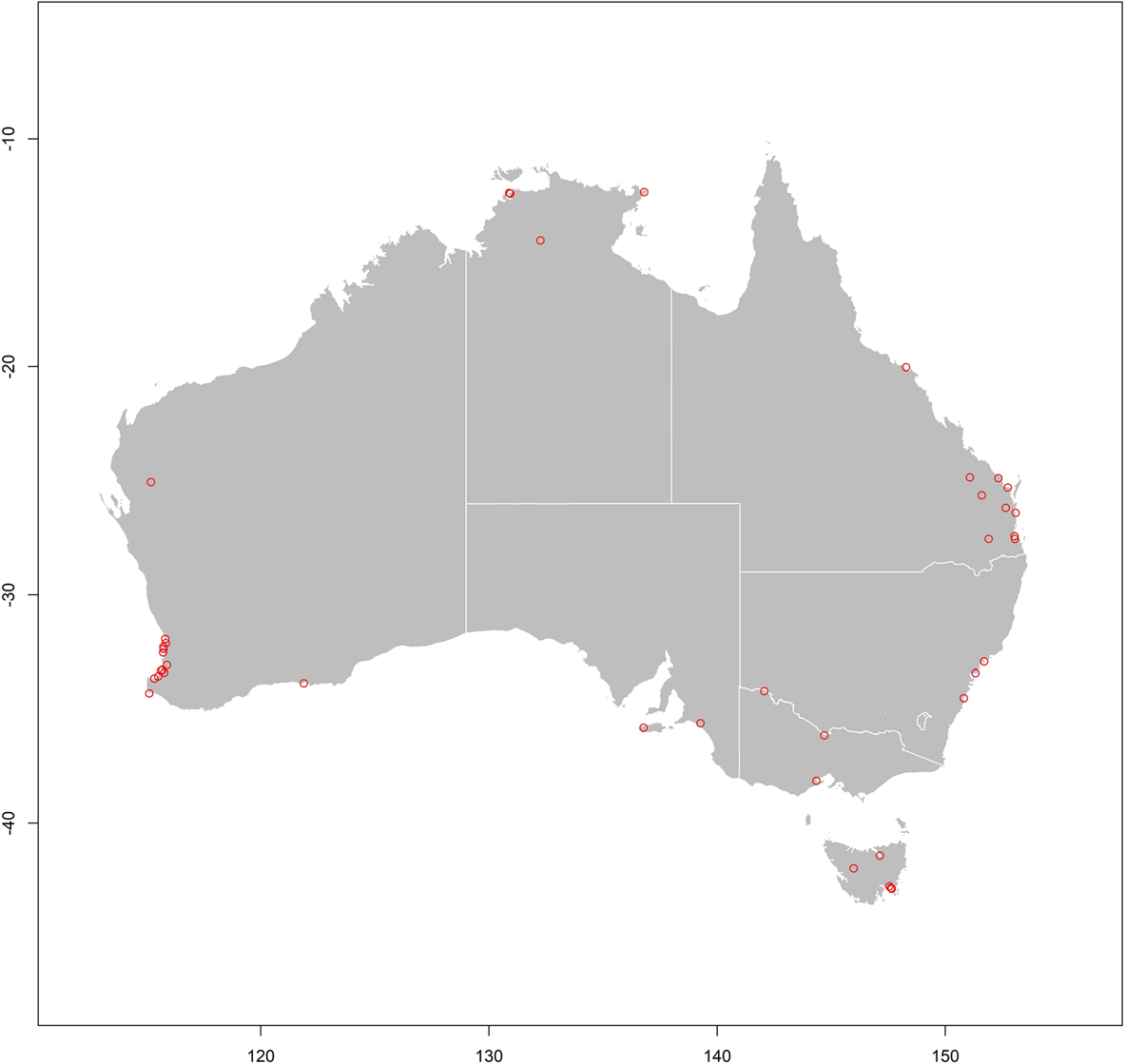
The distribution of the 46 Ross River virus (RRV) epidemics identified through the ProMED system between 1 January, 1996 and 1 July, 2016 in Australia

The geographical distribution of Australian RRV epidemics, as reported by the ProMED electronic surveillance system of the ISID between 1 January, 1996 and 1 July, 2016, is presented in the map in Fig. 1. Large epidemic events were widely distributed around the perimeter of the continent but were concentrated along coastal and peripheral riverine regions.

Climate and hydrological features are mapped in Additional file 1: Figure S1 and Additional file 2: Figure S2, respectively, while the predicted habitat suitability of each RRV host species obtained from ecological niche models is presented in Additional file 3: Figure S3. The characteristics of the niche models used for each species are presented in Additional file 4: Table S1.

**Fig. 2:**
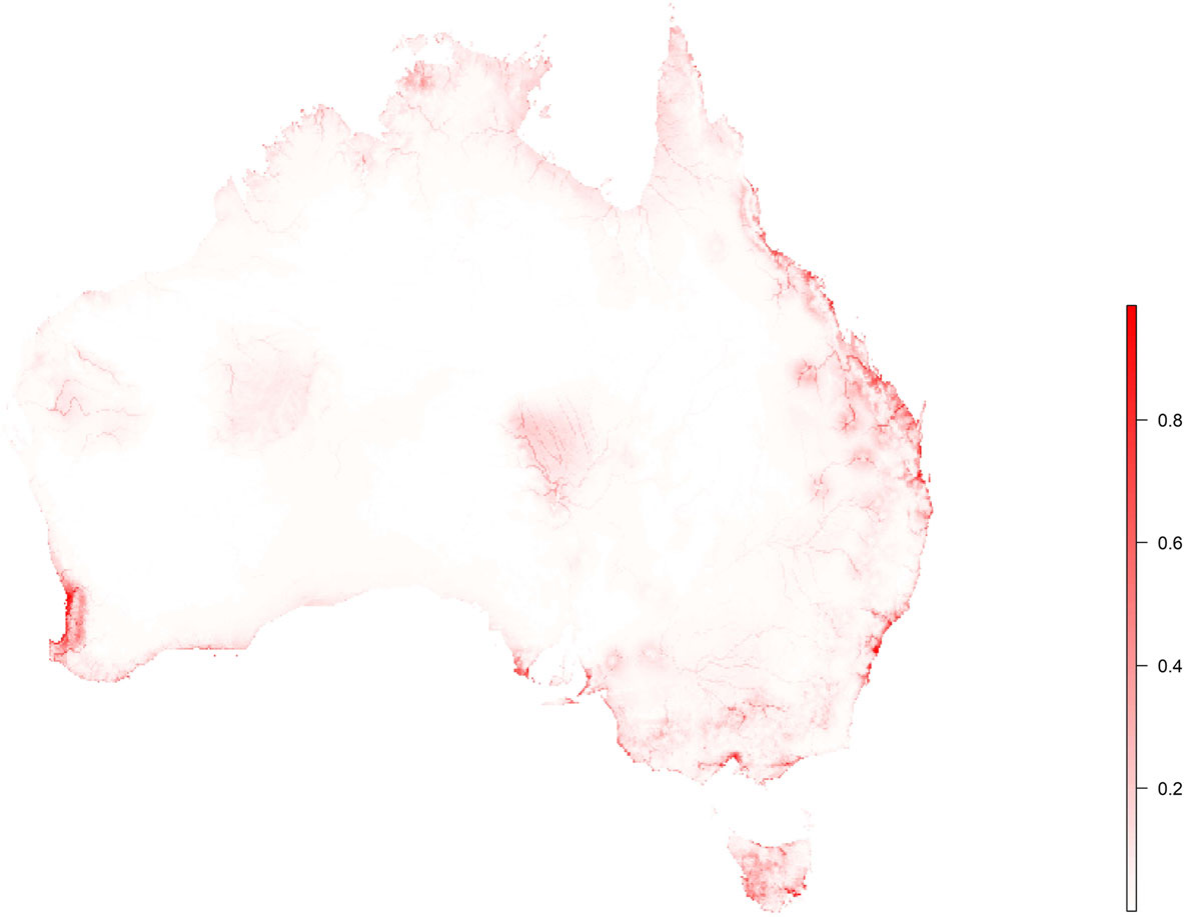
Landscape suitability to Ross River virus epidemics. This risk surface is based on the ecological niche of RRV epidemics as modeled using Maxent

**Fig. 3:**
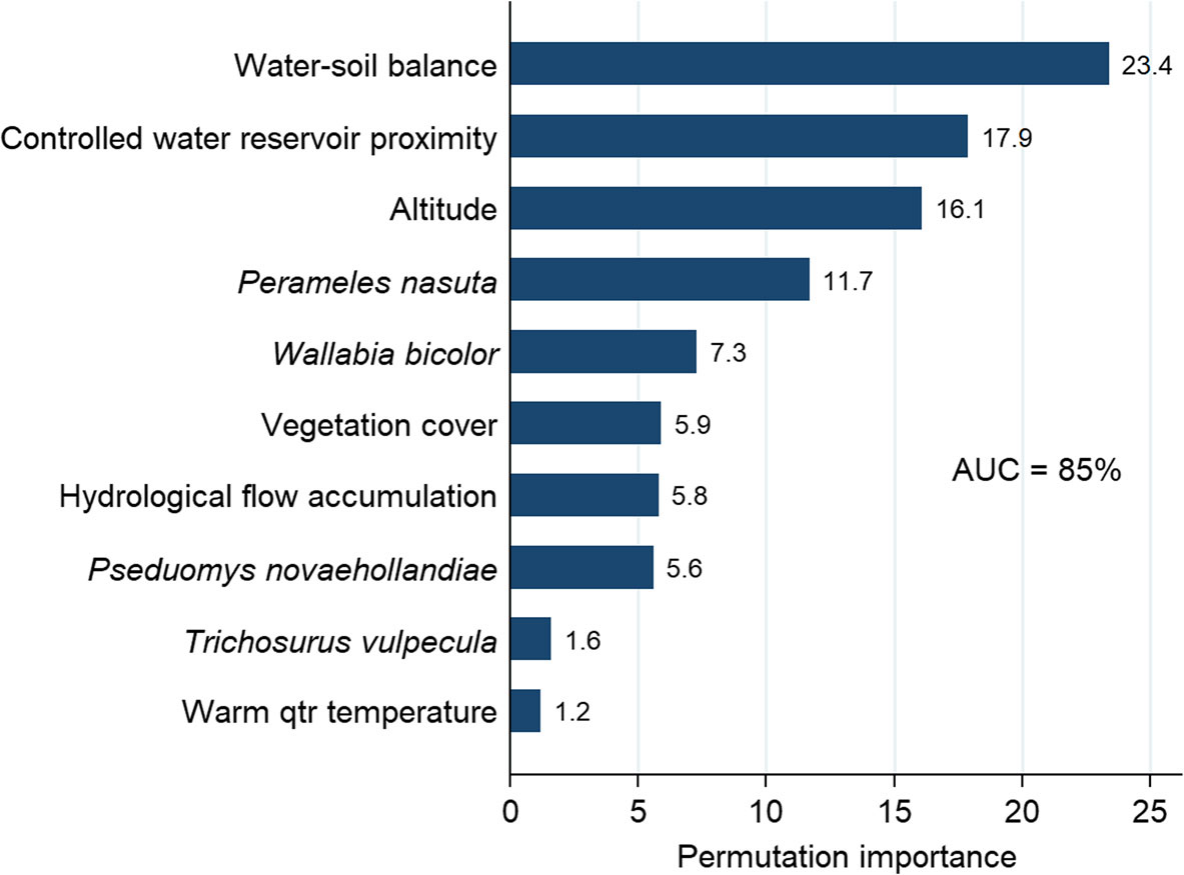
Relative influence of each feature to epidemic RRV landscape suitability as derived from their permutation importance in the Maxent model. Landscape features are ranked from most influential to least with the permutation importance listed at the top of each bar. The area under the curve (AUC) reported as a percentage is also presented to indicate model performance

The landscape suitability of RRV epidemics is presented in Fig. 2 and depicts areas of highest risk for RRV epidemics along the eastern seaboard, consistently in coastal Queensland and the central coast of New South Wales, southern Victoria and the Murray River valley, Tasmania, and far southwestern Western Australia. Figure 3 presents the relative influence of the top ten most influential landscape features and their associated rank in the hierarchy of RRV landscape suitability based on their permutation importance in the Maxent model. These ten features explained 96% of the variation in the data, while the remaining 15 features accounted for only 4% combined. Hydrological features were particularly influential with water-soil balance (permutation importance, PI = 23.4%) and proximity to controlled water reservoirs (PI = 17.9%) ranking first and second, respectively, and flow accumulation (PI = 5.8%) ranking seventh in their overall contribution to the loss function. The predicted ecological niches of four wildlife hosts also proved influential to RRV landscape suitability with an overall contribution to the loss function of 26.2%. Individual contributions were as follows: *P. nasuta* (PI = 11.7%), *W. bicolor* (PI = 7.3%), *P. novaehollandiae* (PI = 5.6%) and *T. vulpecula* (PI = 1.6%). Altitude (PI = 16.1%) and vegetation cover (PI = 5.9%) also demonstrated significant influence on RRV habitat suitability, while mean temperature during the warmest quarter exhibited only modest influence (PI = 1.2%).

**Fig. 4:**
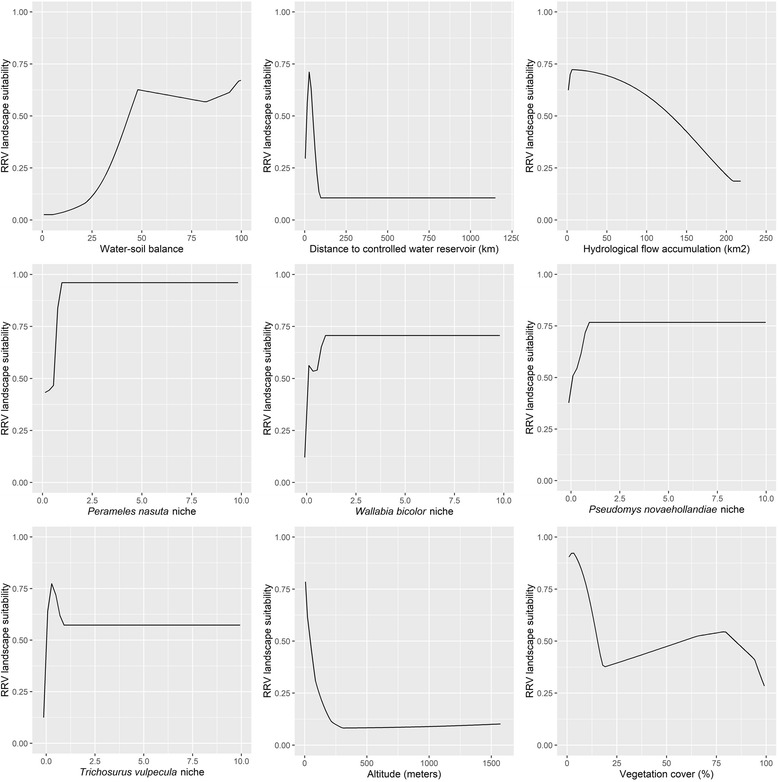
Variable response curves for the hydrological features, the ecological niches of wildlife hosts, altitude and vegetation cover

The response curves showed that closer proximity to controlled water reservoirs was associated with greater RRV landscape suitability (Fig. 4). Increasing soil-water balance in the landscape was strongly associated with increasing risk, but only along the spectrum from the most arid soil-water balance conditions up to a point halfway to the least arid soil-water balance conditions (P-T α ≈ 50%), after which risk did not markedly change. The ecological niche of four wildlife hosts were also associated with RRV risk, with at least a doubling of risk for each species even at modest predicted niche probabilities. The mean AUC for the model was 85%, suggesting good predictive performance.

## Discussion

The current investigation mapped the landscape suitability of large RRV epidemics in anthropogenic environments across the Australian continent based on over 20 years of epidemics reported by the ProMED-mail surveillance system. Moderate soil-water balance, proximity to controlled water reservoirs, altitude, and the ecological niches of *P. nasuta* and *W. bicolor* were key delineators of high-risk landscapes. This is the first study to examine the relationship between RRV epidemics and hydrological dynamics and structure in the landscape. Furthermore, this study considered these and other abiotic features in concert with all known RRV wild mammalian hosts, which is also unique. These findings suggest that RRV epidemic risk is concentrated, but not uniform, around the coastal and near-inland perimeter of Australia. Landscape suitability in this heterogeneous periphery is maximized by the confluence of specific hydrological features and suitable habitat of wildlife hosts.

This study is the first to highlight the importance of this suite of hydrological features in defining landscapes at high risk for RRV epidemics. Moderate soil-water balance and proximity to controlled water reservoirs were the two most influential features of RRV landscape suitability, while hydrological flow accumulation was ranked 7th in importance. Each of these features is important in demarcating unique aspects of the movement of water through the landscape. Controlled reservoirs and dams can be strong mediators of water flow and have the potential to drastically alter natural wetland ecosystems or introduce novel anthropogenic wetland ecosystems [57]. Moreover, in the case of dams specifically, the altered landscape is not limited to the development of controlled water reservoirs upstream of the dam, but also is reflected downstream following the geomorphological reshaping of the riverbed leading to decreased containment of the floodplain [58]. Thus, hydrological restructuring introduces the potential for altered habitats for mosquitoes and reservoir hosts. It is possible that controlled reservoirs and dams have a greater likelihood to have conserved bushland surrounding them that provides suitable refuge for urban wildlife. While proximity to controlled reservoirs has not previously been examined with respect to arboviruses in Australia, it has been shown to increase the risk of mosquito-borne disease transmission in other settings, such as malaria in sub-Saharan Africa [59]. Evidence from the current study may suggest that the placement of water reservoirs could alter wetland ecosystems, or the suitability of the local landscape for reservoir hosts, in ways that enhance RRV landscape suitability.

Water-soil balance, as measured by the P-Tα, demonstrated an interesting relationship with RRV landscape suitability. Suitability was low in highly water-stressed landscapes but increased sharply in landscapes with moderate degrees of water-soil balance, and subsequently reached a threshold of risk at the midpoint of the spectrum. This relationship may highlight the importance of landscapes prone to periodic inundation but which do not experience regular or constant soil-water saturation. This type of soil-water balance would be expected to be particularly relevant to dry inland river flood plains and the *Cx. annulirostris* vector [5]. However, precipitation inundation in coastal estuaries also increases populations of *Ae. vigilax* and *Ae. camptorhynchus* seasonally, rather than tidal inundation alone [5]. An additional driver of the preferred saline habitat of the latter two mosquitoes associated with estuarine wetlands could be the groundwater chemistry associated with reduced evapotranspiration in semi-arid or moderately water-stressed areas adjacent to coastal wetlands [60].

While proximity to lotic systems was not influential to RRV landscape suitability, the geometry of water flow through the landscape was, with areas of lesser mean water flow accumulation associated with increasing risk. This geometry, combined with the overwhelming preponderance of RRV risk experienced at low altitude (Fig. 4), suggests that lowland areas that experience sporadic or seasonal flooding, may be more important than adjacency to a constant lotic flow. This would agree with several local studies, which have found increasing risk associated with precipitation events. Almost all studies examining the association between precipitation and RRV occurrence in humans across three states identified an increase in risk following periods of heavy rain [10–12, 14–17, 61–64]. As mentioned above with respect to water-soil balance, rainfall is likely to modulate the abundance of both inland freshwater and coastal vector mosquitoes. Nevertheless, a recent review [65] found little evidence supporting a significant relationship between flooding and RRV epidemics.

This was the first study to explore the influence of the ecological niche of each known mammalian host on human RRV epidemics. Several studies have identified shedding of RRV in kangaroos and wallabies, with *M. giganteus* and *M. fuliginosus* recognized as potentially important reservoir hosts [7, 8]. These two macropods are generally accepted as driving much of the zoonotic transmission of RRV [5]. However, the current study highlighted the niches of *W. bicolor*, *P. nasuta*, *P. novaehollandiae* and *T. vulpecula* to be most influential to epidemic RRV in anthropogenic landscapes. Identifying these four species as RRV hosts is not novel, and of course cannot be by virtue of their being included in this study, but highlighting their potential importance to epidemic RRV landscape suitability is. Moreover, it is worth noting that the three former species are currently experiencing significant habitat loss due to land development [66–68], whereas the latter species is one that is highly adaptable to human environments [69]. Therefore, these species have the potential to be important bridging hosts in areas of growing human population expansion in lowland wetland ecosystems [9]. There is still much to learn regarding the role of native wildlife in driving risk of RRV transmission in Australia. Beyond the response to infection of individual species, population dynamics of wildlife must also be considered. Surveillance for enzootic RRV activity is restricted to sampling and testing of mosquito populations, whereas suitable strategies to track wildlife serology, as is the case with WNV in North America in which surveillance has targeted wild avian hosts [70], have yet to be developed in Australia. Similarly to WNV in North America [71], spatial and temporal variability in virus circulation in both vector and reservoir hosts, possibly mediated by climatic and abiotic factors, may drive epidemic transmission cycles of RRV but this will require thorough investigation of the viral ecology to determine.

A brief comment is warranted on what appear to be anomalous regions of relatively high risk in the Pilbara region of Western Australia, and the areas around Alice Springs in the Northern Territory (Fig. 2). While these areas appear to stand out in comparison to surrounding areas, they were predicted by the model to have only modest landscape suitability (range 20–40%) relative to much of the surrounding interior, which was predicted close to 0. These areas also correspond to areas of greater water accessibility and higher mammalian biodiversity compared to surrounding desert country. This modest landscape suitability notwithstanding, there were ProMED reports of Ross River epidemics in both the Pilbara [72] and Alice Springs [73], however these could not be included in the analysis because they did not offer sufficient geographical reporting to geolocate the occurrences.

There are some important limitations for which we must provide more explicit discussion. First, temperature and precipitation were aggregated as background composites over a 50-year period, from 1950 to 2000. Therefore, while the 1 km^2^ spatial resolution of this single aggregate measure was reasonably high, it was also temporally coarse, which constrained the analysis as described above in the discussion of weather and climate. Nevertheless, the two quarterly measures of temperature and precipitation are accurate representations of the general climate regimes of the Australian continent, and thus represent a reasonable approach to controlling for background climate. We do recognize, however, our inability to draw any conclusions regarding specific weather events and concede this as an important limitation overall. Moreover, it is worth re-emphasizing that the object of this study was not forecasting, which would require the unavailable weather data at fine temporal (and spatial) scale. We are not trying to predict risk for a specific set of weather conditions or given a particular seasonally-dependent abundance of vector mosquitoes. Rather this study seeks to identify landscapes that are most suitable to epidemic RRV, given hydrological conditions and the ecological niche of key wildlife hosts. For any given year, the actual risk will vary based on local weather events and the subsequent proliferation of mosquitoes. So, while this approach does not predict RRV occurrence given a specific set of weather conditions, it does highlight areas that could be more susceptible to epidemic events if the right weather conditions are present. Secondly, the documented RRV epidemics included in this study are (i) derived from the ProMED surveillance system archive, which may not have captured the reporting of all public health agencies, and (ii) relatively small in number. Therefore, while it was our explicit intention to model the landscape suitability of RRV epidemics within the narrow scope of anthropogenic environments, these data may not be representative of the total epidemic events occurring within this landscape cross-section of Australia over the time period under study. It is also important to note that the landscape epidemiology of epidemic RRV is not expected to operate within the same ecological phenomena as sporadic RRV. As such, conclusions drawn from the current study necessarily apply only to epidemic RRV in anthropogenic landscapes. Thirdly, the ecological niche modeling of the reservoir species is subject to potential spatial sampling bias, in that specimens are more likely to be sampled in those areas that are more accessible to sampling. While we cannot eliminate such bias entirely, we did mitigate its effects by sampling background points proportional to the human footprint, a rich proxy for accessibility. Thus, for each species, we were able to model the niche itself rather than the artefactual phenomenon of observability. Fourth, mosquito populations were not included in the RRV landscape suitability model. While vector mosquito species (*Ae. vigilax*, *Ae. camptorhynchus* and *Cx. annulirostris*) are available in GBIF the observations are obtained across broad spans of time (1950–1995) and while this corresponds well to the time period over which climate is measured (1950–2000), it is unlikely that this metric can adequately model the sparse mosquito data across such large periods of time. Following from these limitations, the findings do not posit a definitive understanding of epidemic RRV. They may extend the understanding of the landscape epidemiology of RRV in humans by highlighting specific features that are relevant to RRV epidemics. However, it is essential that this model be validated against new data as they become available from better field investigations in broad and diverse environmental settings across Australia. Finally, because this is an observational study, direct causal interpretation of the associations between RRV epidemics and landscape factors are not inferred. The associations presented here may suggest relationships among landscape features and RRV epidemics, but they do not suggest causality. More definitive causal inference will require direct measurement of the biotic, abiotic and human social landscapes where RRV epidemics emerge and where they do not, preferably at high spatial resolution and in real time. This will require extensive field investigation incorporating human and animal sampling, multi-species mosquito surveillance, rich habitat description, and fine-scaled weather time-series.

## Conclusion

In conclusion this study found that features mediating the movement of water through the landscape and the ecological niche of wildlife hosts promoted landscapes suitable to RRV epidemics in anthropogenically impacted environments. Taken together these features help to delineate epidemic RRV risk in Australia, and thus may help direct geographically targeted vector and wildlife surveillance within and across state boundaries, and in concert with human syndromic surveillance. Careful monitoring of key wildlife populations and protection of their habitat in peri-urban spaces may be warranted.

## Additional files

Additional file 1: **Figure S1.** The distribution of mean precipitation during the driest and wettest quarters and mean temperature during the coldest and warmest quarters across Australia. (TIFF 2959 kb)

Additional file 2: **Figure S2.** Vegetation cover and hydrological distribution maps. Each pixel the upper right coastal distance panel represents the distance between that 1 km^2^ area and the nearest pixel containing that water feature. The bottom left panel represents water stress, or the soil-water balance, as measured by the Priestley-Taylor α coefficient. The bottom right panel represents flow accumulation, which measures the number of upstream km^2^ that drain into each 1 km^2^. (TIFF 5882 kb)

Additional file 3: **Figure S3.** The distributions of habitat suitability of the mammalian hosts, as predicted by Maxent models of their ecological niches. The top row of highlighted maps represents those host species that were influential to RRV epidemic landscape suitability. (TIFF 2044 kb)

Additional file 4: **Table S1.** Description of the ecologic niche models for each of the predicted species distributions for mammalian hosts. (DOCX 15 kb)

## Abbreviations

API: Application programming interface
AUC: Area under the curve
GBIF: Global Biodiversity Information Facility;
MGVF: Maximum Green Vegetation Fraction
MODIS: Moderate-Resolution Imaging Spectroradiometer
RRV: Ross River virus
SEDAC: Socioeconomic Data and Applications Center
WNV: West Nile virus
