# Supplementary figures and images for: Hydrological features and the ecological niches of mammalian hosts delineate elevated risk for Ross River virus epidemics in anthropogenic landscapes in Australia
Source: Parasit Vectors. 2018 Mar 20;11:192. doi: 10.1186/s13071-018-2776-x (PMC5859420; doi:10.1186/s13071-018-2776-x)

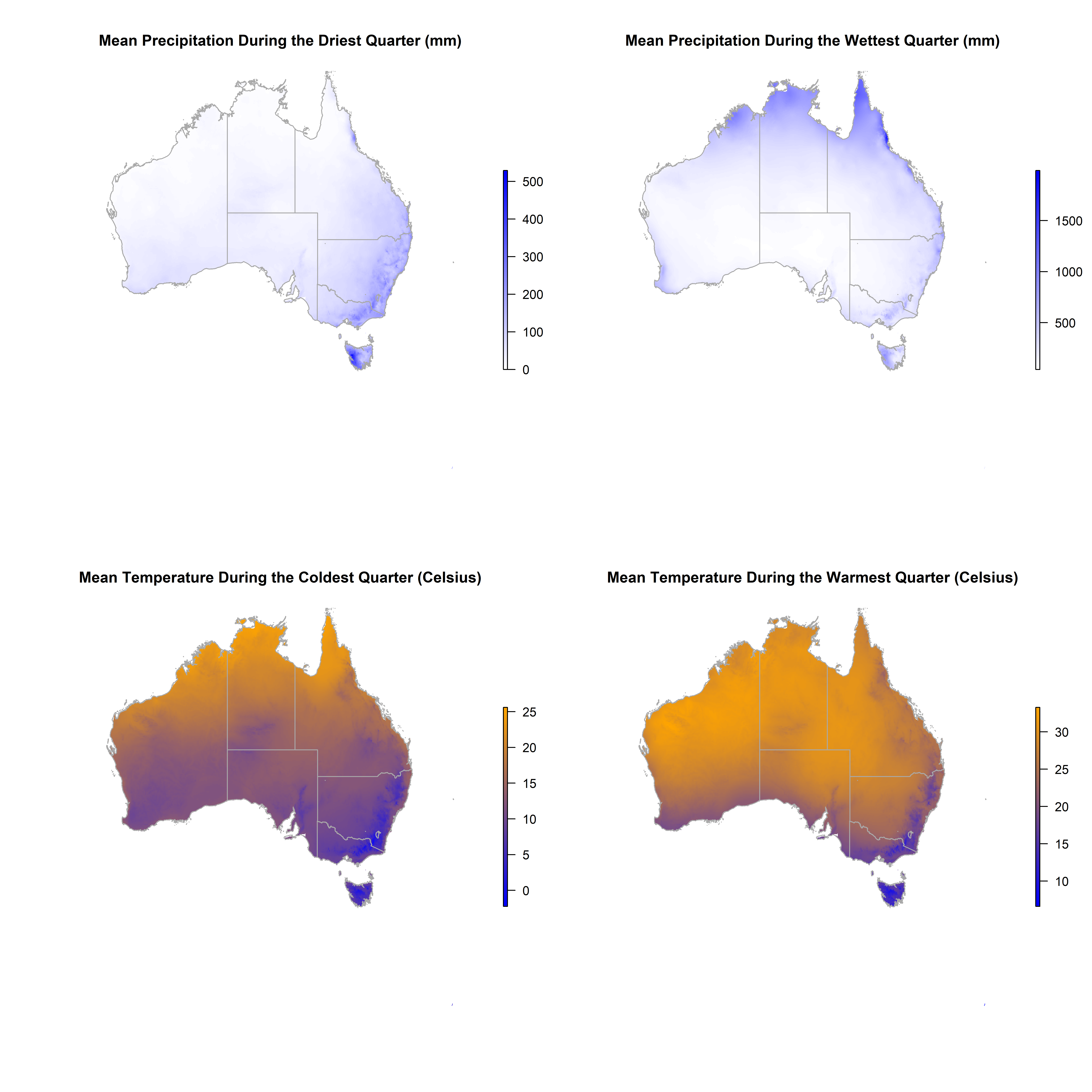

Supplement: Supplementary file 1 — Figure S1. The distribution of mean precipitation during the driest and wettest quarters and mean temperature during the coldest and warmest quarters across Australia. (TIFF 2959 kb) [file 13071_2018_2776_MOESM1_ESM.tiff]

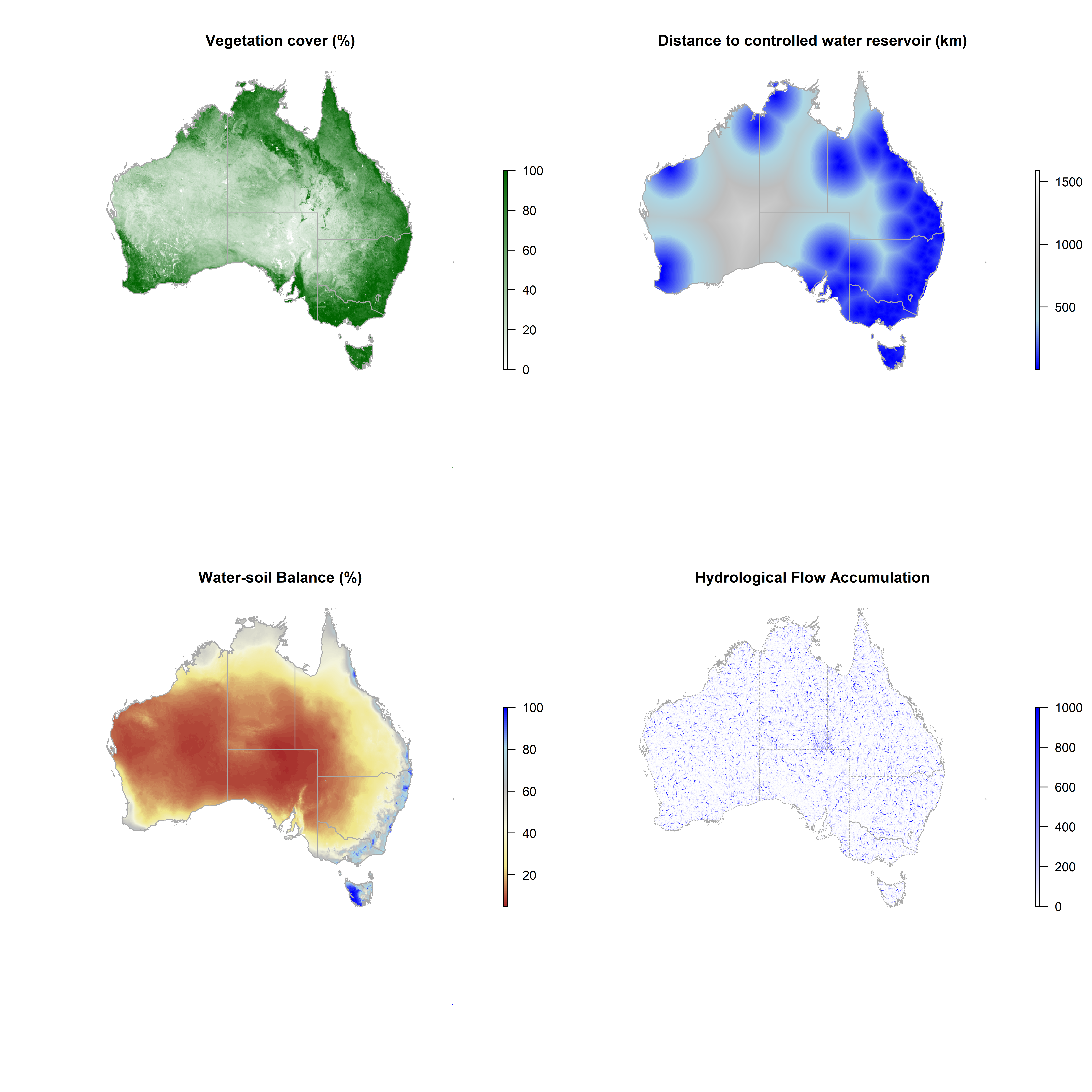

Supplement: Supplementary file 2 — Figure S2. Vegetation cover and hydrological distribution maps. Each pixel the upper right coastal distance panel represents the distance between that 1 km2 area and the nearest pixel containing that water feature. The bottom left panel represents water stress, or the soil-water balance, as measured by the Priestley-Taylor α coefficient. The bottom right panel represents flow accumulation, which measures the number of upstream km2 that drain into each 1 km2. (TIFF 5882 kb) [file 13071_2018_2776_MOESM2_ESM.tiff]

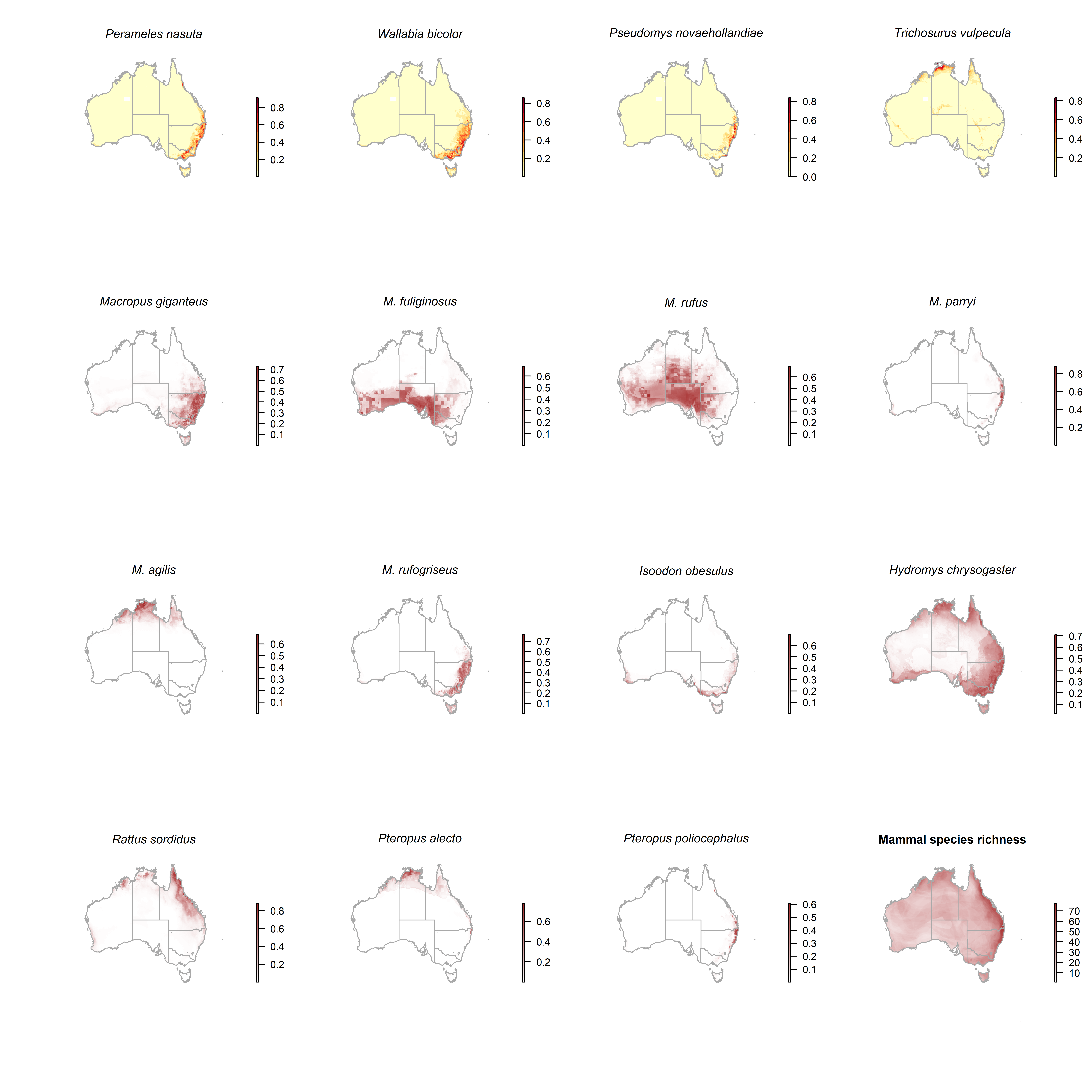

Supplement: Supplementary file 3 — Figure S3. The distributions of habitat suitability of the mammalian hosts, as predicted by Maxent models of their ecological niches. The top row of highlighted maps represents those host species that were influential to RRV epidemic landscape suitability. (TIFF 2044 kb) [file 13071_2018_2776_MOESM3_ESM.tiff]
