# Supplementary material for: Hydrological features and the ecological niches of mammalian hosts delineate elevated risk for Ross River virus epidemics in anthropogenic landscapes in Australia
Source: Parasit Vectors. 2018 Mar 20;11:192. doi: 10.1186/s13071-018-2776-x (PMC5859420; doi:10.1186/s13071-018-2776-x)
Supplement: Supplementary file 4 — Table S1. Description of the ecologic niche models for each of the predicted species distributions for mammalian hosts. (DOCX 15 kb) [file 13071_2018_2776_MOESM4_ESM.docx]

Table 1. Description of the ecologic niche models for each of the predicted species distributions for mammalian hosts. For each species model the area under the curve (AUC) is presented to describe Maxent model performance, and the ranking of the top five landscape features defining the ecologic niche are listed with their respective relative influences reported as a percentage in parentheses. The rank order is determined by the magnitude of the permutation importance. Precipitation (Precip) during the driest and wettest quarters (qtr), and temperature (Temp) during the warmest and coldest quarters represent the mean measurements during these quarters over the period 1950 to 2000. Vegetation cover is the mean green vegetation fraction from 2001 to 2012. Human migration is the change in human population between 1990 and 2000 per 1 km^2^.

| Predicted SDM | AUC | Rank 1 (%) | Rank 2 (%) | Rank 3 (%) | Rank 4 (%) | Rank 5 (%) |
| --- | --- | --- | --- | --- | --- | --- |
| *Macropus giganteus* | 89% | Precip, driest qtr (76.0) | Temp, warmest qtr (8.0) | Human migration (5.9) | Vegetation cover (4.9) | Temp, coldest qtr (2.8) |
| *Macropus fuliginosus* | 83% | Precip, driest qtr (43.4) | Temp, coldest qtr (17.1) | Temp, warmest qtr (15.7) | Human migration (14.5) | Vegetation cover (5.0) |
| *Macropus rufus* | 83% | Precip, wettest qtr (38.3) | Precip, driest qtr (26.2) | Human migration (10.7) | Temp, coldest qtr (9.4) | Temp, warmest qtr (8.0) |
| *Macropus robustus* | 87% | Temp, coldest qtr (38.6) | Human migration (22.9) | Temp, warmest qtr (13.4) | Precip, driest qtr (9.2) | Vegetation cover (8.1) |
| *Macropus agilis* | 97% | Precip, wettest qtr (73.4) | Temp, coldest qtr (13.6) | Human migration (6.2) | Temp, warmest qtr (3.4) | Vegetation cover (3.0) |
| *Macropus rufogriseus* | 94% | Precip, driest qtr (35.5) | Precip, wettest qtr (27.5) | Temp, warmest qtr (14.5) | Human migration (8.2) | Vegetation cover (7.9) |
| *Macropus parryi* | 97% | Precip, wettest qtr (60.9) | Temp, coldest qtr (29.6) | Precip, driest qtr (7.1) | Temp, warmest qtr (1.9) | Vegetation cover (0.6) |
| *Wallabia bicolor* | 94% | Precip, driest qtr (68.9) | Temp, warmest qtr (12.0) | Precip, wettest qtr (7.5) | Vegetation cover (6.8) | Temp, coldest qtr (2.8) |
| *Trichosurus vulpecula* | 95% | Precip, wettest qtr (33.9) | Temp, coldest qtr (30.0) | Precip, driest qtr (14.0) | Human migration (10.7) | Temp, warmest qtr (7.0) |
| *Isoodon obesulus* | 91% | Temp, warmest qtr (70.5) | Precip, wettest qtr (18.6) | Temp, coldest qtr (4.7) | Human migration (3.0) | Precip, driest qtr (1.9) |
| *Perameles nasuta* | 95% | Precip, driest qtr (66.5) | Precip, wettest qtr (25.3) | Temp, warmest qtr (7.2) | Vegetation cover (0.8) | Temp, coldest qtr (0.2) |
| *Pteropus poliocephalus* | 95% | Precip, wettest qtr (66.1) | Precip, driest qtr (24.8) | Temp, warmest qtr (6.6) | Temp, coldest qtr (1.5) | Human migration (0.8) |
| *Pteropus alecto* | 98% | Temp, coldest qtr (47.8) | Precip, wettest qtr (33.1) | Precip, driest qtr (13.3) | Temp, warmest qtr (3.7) | Human migration (2.0) |
| *Hydromys chrysogaster* | 72% | Precip, driest qtr (43.0) | Temp, warmest qtr (28.2) | Precip, wettest qtr (9.2) | Human migration (7.9) | Temp, coldest qtr (6.8) |
| *Rattus sordidus* | 94% | Precip, wettest qtr (67.1) | Temp, warmest qtr (25.2) | Vegetation cover (2.8) | Temp, coldest qtr (2.6) | Precip, driest qtr (2.2) |
| *Pseudomys novaehollandiae* | 97% | Precip, driest qtr (62.4) | Temp, warmest qtr (20.5) | Precip, wettest qtr (6.1) | Temp, coldest qtr (5.1) | Vegetation cover (3.7) |
